# Identification and characterization of BATF3 as a context-specific coactivator of the glucocorticoid receptor

**DOI:** 10.1371/journal.pone.0181219

**Published:** 2017-07-14

**Authors:** Petra Birth, Stefanie Schöne, Ulrich Stelzl, Sebastiaan H. Meijsing

**Affiliations:** 1 Max Planck Institute for Molecular Genetics, Ihnestraße 63–73, Berlin, Germany; 2 Department of Pharmaceutical Chemistry, Institute of Pharmaceutical Sciences, University of Graz and BioTechMed-Graz, Universitätsplatz 1, Graz, Austria; Universite de Geneve, SWITZERLAND

## Abstract

The ability of the glucocorticoid receptor (GR) to regulate the transcriptional output of genes relies on its interactions with transcriptional coregulators. However, which coregulators are required for GR-dependent activation is context-dependent and can be influenced by the sequence of the DNA bound by GR and by the nature of the GR isoform responsible for the regulation of a gene. Here, we screened for GR-interacting proteins for which the interaction signal differed between two GR isoforms GRα and GRγ. These isoforms diverge by a single amino acid insertion in a domain, the lever arm, which adopts DNA sequence-specific conformations. We identify Basic Leucine Zipper ATF-Like Transcription Factor 3 (BATF3), an AP-1 family transcription factor, as a GR coregulator whose interaction with GR is modulated by the lever arm. Further, a combination of experiments uncovered that BATF3 acts as a gene-specific coactivator of GR whose coactivator potency is influenced by the sequence of the GR binding site. Together, our findings suggest that GR isoform and the sequence of GR binding site influence the interaction of GR with BATF3, which might direct the assembly of gene-specific regulatory complexes to fine-tune the expression of individual GR target genes.

## Introduction

The binding of glucocorticoid hormones to the glucocorticoid receptor (GR) initiates a cascade of events resulting in changes in the expression level of a cell type-specific subset of genes. These events include translocation of GR to the nucleus, DNA binding and interactions of GR with broad spectrum of coregulators that play a critical role in GR-dependent transcriptional regulation [1]. Coregulators can be grouped into two classes: (i) coactivators that increase GR’s ability to activate transcription and (ii) corepressors that mediate transcriptional repression. How these coregulators contribute to GR-dependent gene regulation varies. For instance, GR can interact directly with components of the basal transcription machinery [2,3] or with components of the mediator complex [4], which in turn recruits RNA polymerase II. Additionally, GR can influence transcript levels by interacting with proteins that regulate transcriptional elongation [5,6]. Other coregulators recruited by GR influence transcription indirectly by remodeling the chromatin [7] or by acting as enzymes that add or remove posttranslational modifications of histones [8] or of RNA polymerase II [9].

Notably, individual GR target genes in a given cell type may rely on interactions with distinct coregulators [6,10–12]. Accordingly, the GR surfaces that interact with these coregulators are also required in a gene-specific manner [13]. These observations argue that different regulatory assemblies act at individual GR target genes. Such gene-specific assemblies might in turn play a role in fine-tuning the expression level of individual GR target genes in a cell. Several factors are implicated in directing the assembly of distinct regulatory complexes and in modulating the transcriptional output of individual GR target genes. These factors include posttranscriptional modifications of GR [14] and the presence or absence of binding sites for other transcription factors at GR-bound loci [15]. The sequence composition of the core DNA binding site of GR can also modulate GR’s activity [16–18]. In several cases, these sequence-induced changes in GR activity cannot be explained by differences in GR occupancy [16] arguing that the modulation is a consequence of events downstream of DNA binding. Accordingly, the sequence of the GR binding sequence (GBS) induces conformational changes in the DNA binding domain and influences which functional domains are required for GR-dependent transcriptional activation [17,18]. This suggests that GBS variants nucleate the assembly of distinct regulatory complexes and accordingly, the effect of knockdown of the GR coregulator BRM, the ATPase subunit of the SWI/SNF chromatin remodeling complex, is GBS-specific [17].

**Fig 1:**
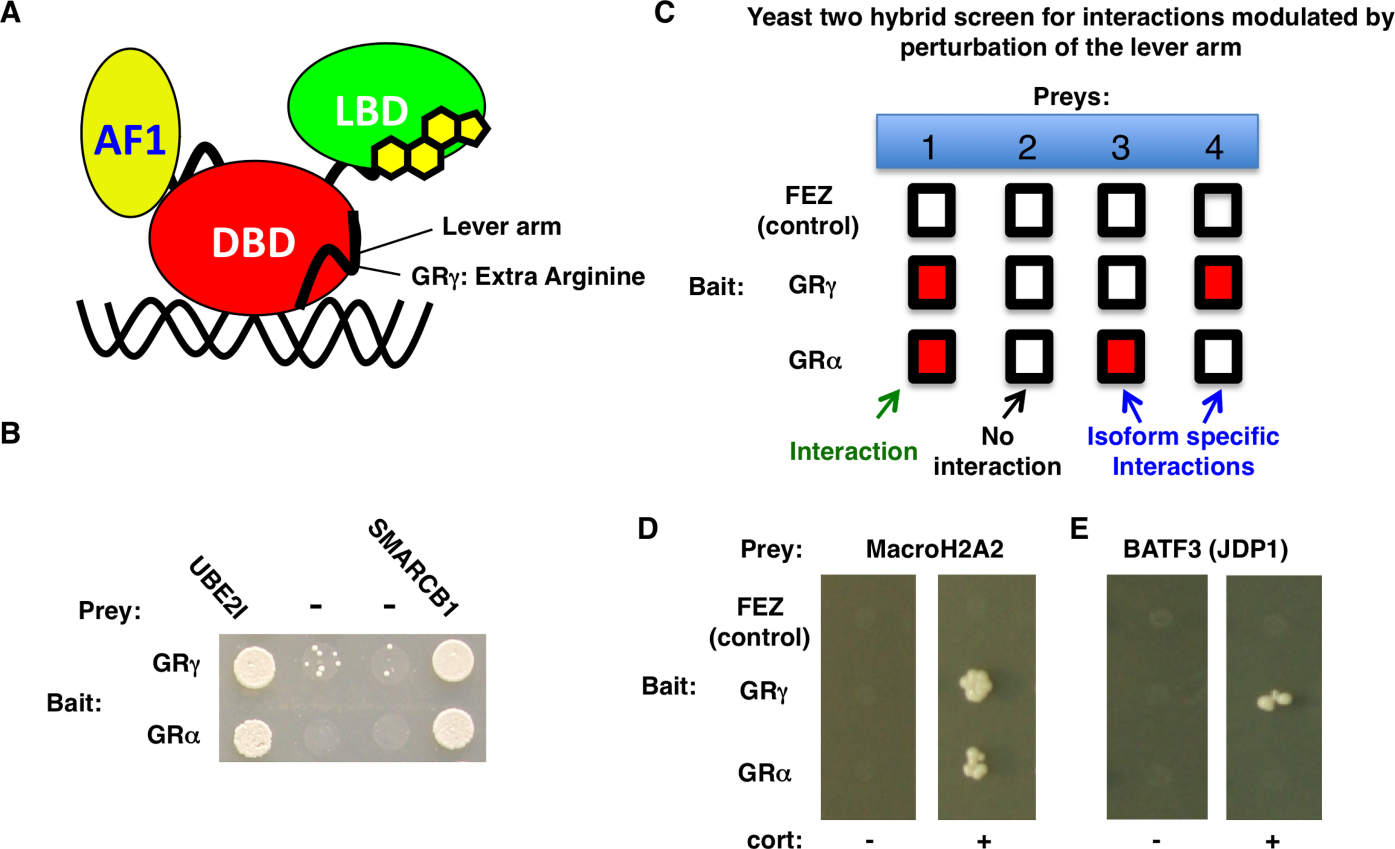
Y2H screen for GR isoform-specific interactions. (a) Domain structure of GR highlighting the ligand binding domain (LBD), Activation Function 1 (AF1) and the DNA binding domain (DBD) which includes the lever arm that diverges between GRα and GRγ. (b) Selective Y2H plate containing 1 μM desoxycorticosterone. Colonies indicate that both GRα and GRγ interact with known GR interaction partners UBE2I and SMARCB1 whereas GRγ shows some autoactivation at this hormone concentration (prey -: empty prey plasmid) (c) Schematic representation of the screen. Isoform-specific interactions are identified by comparing pairwise interactions between each prey with either GRα, GRγ, or FEZ (control for specificity of the interaction) as bait. Red marked boxes represent interactions, white boxes a lack of interaction between bait and prey. (d) Selective Y2H plates either without (left) or with (right) hormone (300 nM desoxycorticosterone (cort)). Colonies indicate an interacting bait-prey pair between the bait as indicated and macroH2A2. (e) Same as for (d) except that prey is BATF3 (JDP1).

Gene-specific coregulator requirements and responses to glucocorticoid signaling can also be facilitated by distinct GR isoforms that arise from alternative splicing and alternative translational initiation events [19,20]. For example, translational isoforms of GR regulate different sets of genes and recruit distinct coregulators [21]. Similarly, two naturally occurring isoforms, GRα and GRγ, regulate only partially overlapping sets of genes [22,23]. GRα and GRγ differ by a single amino acid insertion in the lever arm, a domain that adopts DNA sequence specific conformations (Fig 1A). The lever arm insertion alters transcriptional regulation by GR in a context-specific manner through two mechanisms: Differential DNA binding and altered communication between GR domains [22] which might result in the assembly of distinct regulatory complexes.

Based on the findings described above, we reasoned that perturbation of the lever arm either by insertion of an arginine due to alternative splicing or by GBS-induced structural changes might modulate the interaction of GR with certain coregulators. To identify such coregulators, we performed a systematic yeast two-hybrid screen where we compared the interaction profile of GRα and GRγ with a matrix of ~ 12000 full-length human proteins [24]. This screen resulted in the identification of Basic Leucine Zipper ATF-Like Transcription Factor 3 (BATF3), an AP-1 family transcription factor, as a GR coactivator whose interaction with GR is modulated by the lever arm. Further, a combination of experiments uncovered that BATF3 acts as a gene-specific coactivator of GR whose regulatory potential is modulated by the sequence of the DNA binding site.

## Results

### Identification of BATF3 as a GR isoform-specific interaction partner

To identify coregulators whose interaction with GR is modulated by perturbation of the lever arm, we set out to compare binary protein:protein interactions for GRα and GRγ using the yeast two-hybrid (Y2H) system. Initial experiments showed high levels of auto-activation for both GR isoforms in the presence of hormone. To circumvent this problem, we mutated three residues (E219K/F220L/W234R) in the amino-terminal activation domain of both GR isoforms. Consistent with previous studies [4], these mutations markedly reduced hormone-dependent auto-activation by GR in the Y2H assay although some background growth was still observed at high hormone concentrations, especially for the GRγ isoform (Fig 1B). However, the mutated GRα and GRγ isoforms retained their ability to interact with known GR cofactors SMARCB1 [25] and UBE2I [26] (Fig 1B). Next, we systematically analyzed the Y2H interaction profile for GRα and GRγ with a matrix of ~12000 full-length human proteins [24] both in the presence and absence of hormone (Fig 1C). The Y2H screen and subsequent re-test uncovered several novel hormone-dependent interactions that were shared between the GRα and GRγ isoforms, for example with the histone variant macroH2A2 which interacted with both GRα and GRγ in the initial screen and in 4 out of 4 subsequent re-tests (Fig 1D). In addition, the initial Y2H screen identified a GRγ-specific interaction between Basic Leucine Zipper ATF-Like Transcription Factor 3 (BATF3) and GRγ (Fig 1D). Subsequent re-tests confirmed the isoform-specific interaction between BATF3 and GRγ in 3 out of 4 cases with one re-test showing no interaction between BATF3 and either isoform. BATF3 is also known as JDP1, and is a close homolog of the Jun dimerization protein-2 (JDP2), which is a known co-activator of GR [27]. Interestingly, JDP2 interacts with the DNA binding domain (DBD) of GR [27]. Similarly, both BATF3 and JDP2 interact with the DBD of the progesterone receptor (PR) [28], which shares 88% amino acid sequence identity with the DBD of GR. Furthermore, nuclear magnetic resonance (NMR) studies have shown that JDP2 interacts with helix 3 of the DNA binding domain of PR and of particular interest for to this study with the lever arm of PR [29], which is identical in sequence to the lever arm of GR. Together, these findings suggest that the GR isoform-specific interaction with BATF3 could be explained by a direct perturbation of the interaction interface between GR and BATF3 as a consequence of insertion of an extra amino acid in the lever arm. Therefore, we decided to focus our attention on this protein.

**Fig 2:**
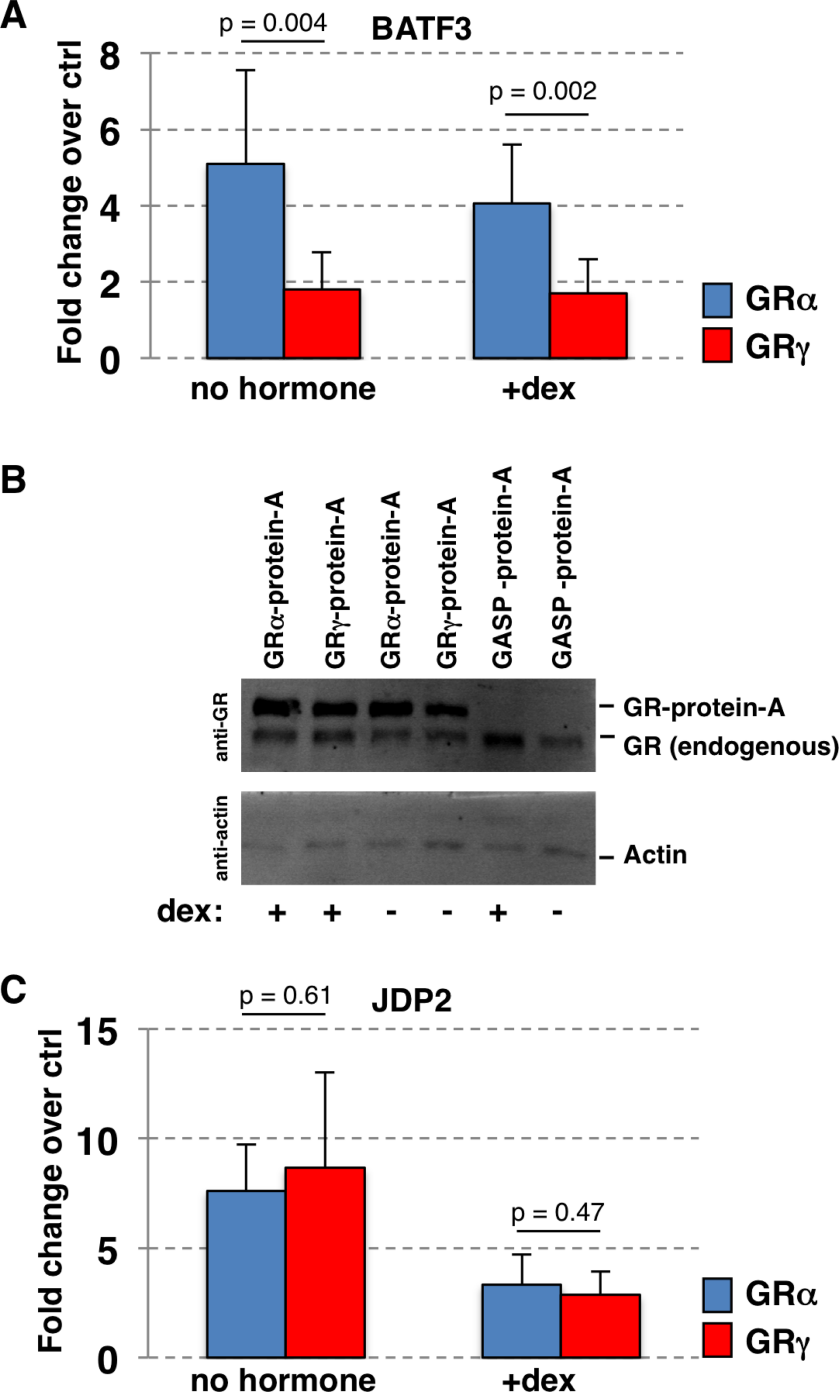
Co-IP assays comparing protein:protein interactions between GRα and GRγ. (a) Fold change over control co-IPs (protein A only) for the binding of the GR isoform as indicated with BATF3 (fused to firefly luciferase). Averages ± standard deviation from at least triplicate transfections are shown for experiments done in either the presence (1 μM dexamethasone (dex)) or absence (no hormone) of GR ligand. The p-values were calculated using a two-tailed Student’s t-test (n = 6). (b) Western blot analysis of the lysates used for co-IP experiments showing similar levels for GRα- and GRγ-protein A fusion proteins. Lysates were from cells transfected with the fusion protein as indicated; cells were either treated with dexamethasone (1 μM, “+”) or with ethanol vehicle (“-“). (c) Same as for (a) except that the interaction between JDP2 and GRα or GRγ was quantified. The p-values were calculated using a two-tailed Student’s t-test (n = 9).

To validate the isoform-specific Y2H interaction between GR and BATF3, we analyzed their interaction in luciferase based co-IP assays [30] in mammalian cells both in the presence and absence of hormone. In these assays, the amount of luciferase-BATF3 fusion protein co-IPed with either GRα or GRγ was measured in a luciferase assay and compared between the two GR isoforms. Consistent with the Y2H assays, we found that the interaction between BATF3 and GR was isoform-specific (Fig 2A). In contrast with the Y2H experiments however, this time the luciferase signal for the interaction was higher for GRα than for GRγ and was observed both in the presence and absence of hormone (Fig 2A). Notably, the protein A tagged GRα and GRγ proteins used in the co-IPs were expressed at comparable levels, arguing that the observed isoform-specific interaction signal is not a simple consequence of differences in expression levels (Fig 2B).

Given the high level of sequence similarity between BATF3 and JDP2, we also set out to test if JDP2’s interaction with GR is isoform-specific. Interestingly however, we found that the signal for the interaction with JDP2 is similar for GRα and GRγ (Fig 2C). Together, the Y2H and co-IP experiments indicate that the interaction between GR and BATF3 can be modulated by perturbation of the lever arm. Depending on the assay, addition of an arginine in the lever arm of GR can either weaken (co-IP) or strengthen (Y2H) the interaction.

### BATF3 is a context-specific GR co-activator

**Fig 3:**
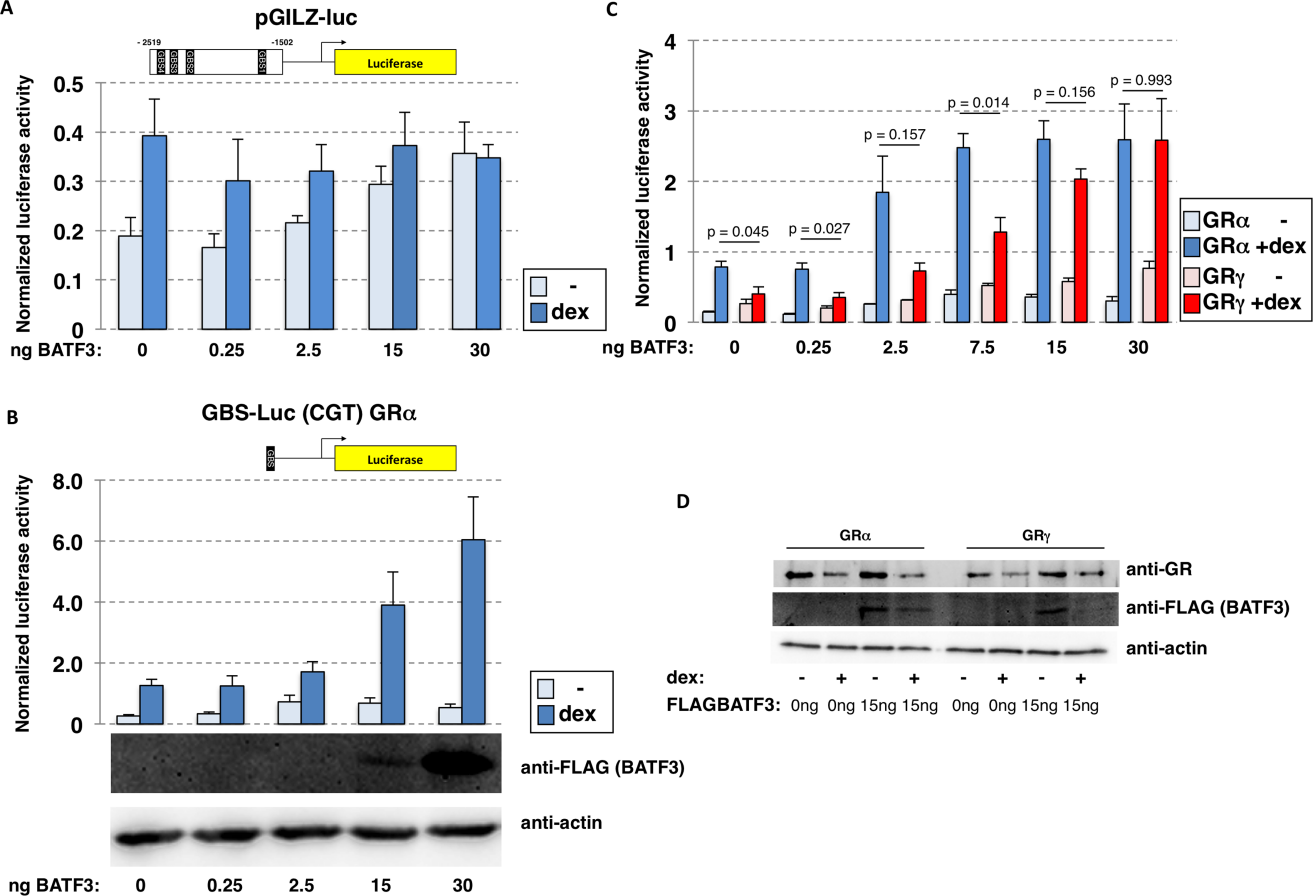
BATF3 is a context-specific co-activator of GR. (a) U2OS cells were cotransfected with an expression construct for GRα, a luciferase reporter encompassing a minimal promoter and a ~1kb GR region derived from the GR-responsive *GILZ* gene and increasing amounts of an expression construct for FLAG-tagged BATF3. Average normalized luciferase activity ± S.E.M. (n = 3) is shown for cells treated overnight with either 1 μM dexamethasone (dex) or ethanol (-) as vehicle control. (b, top) Same as for (a) except that a different luciferase reporter containing a minimal promoter and the CGT GBS was used. (b, bottom) Western blot analysis of dexamethasone-treated cells transfected with increasing amounts of FLAG-tagged BATF3 expression construct. Expression of FLAG-tagged BATF3 and actin (loading control) are shown. (c) U2OS cells stably expressing comparable amounts of either GRα or GRγ were transfected with the CGT luciferase reporter and increasing amounts of FLAG-tagged BATF3 expression construct. Average normalized luciferase activity ± S.E.M. (n = 3) is shown for cells treated overnight with either 1 μM dexamethasone (dex) or ethanol (-) as vehicle control. The p-values were calculated using a two-tailed Student’s t-test. (d) Western blot analysis of cells U2OS cells stably expressing comparable amounts of either GRα or GRγ that were transfected as described for with 15ng FLAG-tagged BATF3 expression construct and treated overnight with either 1 μM dexamethasone (+) or ethanol (-) as vehicle control. Expression of GR, FLAG-tagged BATF3 and actin (loading control) are shown.

To test the effect of BATF3 on GR-dependent transcriptional regulation, we assayed the effect of BATF3 overexpression on the GRα-dependent regulation of the well-characterized *GILZ* luciferase reporter [31]. This reporter contains an approximately 1kb region, which contains multiple GBSs and is derived from a genomic region near the GR target gene *GILZ*. We found that hormone-independent (basal) reporter activity slightly increased with increasing amounts of BATF3 expression construct (Fig 3A). In contrast, increasing amounts of BATF3 did not result in a marked change in the hormone-induced levels of the *GILZ* reporter (Fig 3A) arguing that BATF3 does not act as a GR coregulator for the *GILZ* reporter. To test if BATF3 might influence GR in another context, we next assayed the effect of BATF3 overexpression on a luciferase reporter consisting of a single GBS upstream of a minimal promoter [17]. Contrary to our findings with the *GILZ* reporter, this time we observed a marked increase in the hormone-induced levels. For example, the hormone-induced level of reporter activity at the highest BATF3 amount was ~ 5 fold higher than the level observed in the absence of BATF3 overexpression (Fig 3B).

To compare the effect of overexpression of BATF3 between GRα and GRγ, we transfected U2OS cell lines stably expressing similar amounts of either GR isoform (Fig 3D) with the single GBS reporter. Similar to our observations with transiently transfected GRα, BATF3 overexpression resulted in increased transcriptional activation by both GRα and GRγ expressing U2OS cells (Fig 3C) arguing that BATF3 can function as coactivator for both GR isoforms. Matching our previous observations [17], activation of the single GBS reporter was weaker for GRγ than for GRα in the absence of BATF3 overexpression (Fig 3C). However, the hormone-induced levels of reporter activity became comparably for GRα and GRγ when cells were transfected with high doses of BATF3 construct (Fig 3C), which might compensate for the lower affinity of GRγ for BATF3, and possibly other coactivators, and GRγ’s weaker ability to activate the reporter.

**Fig 4:**
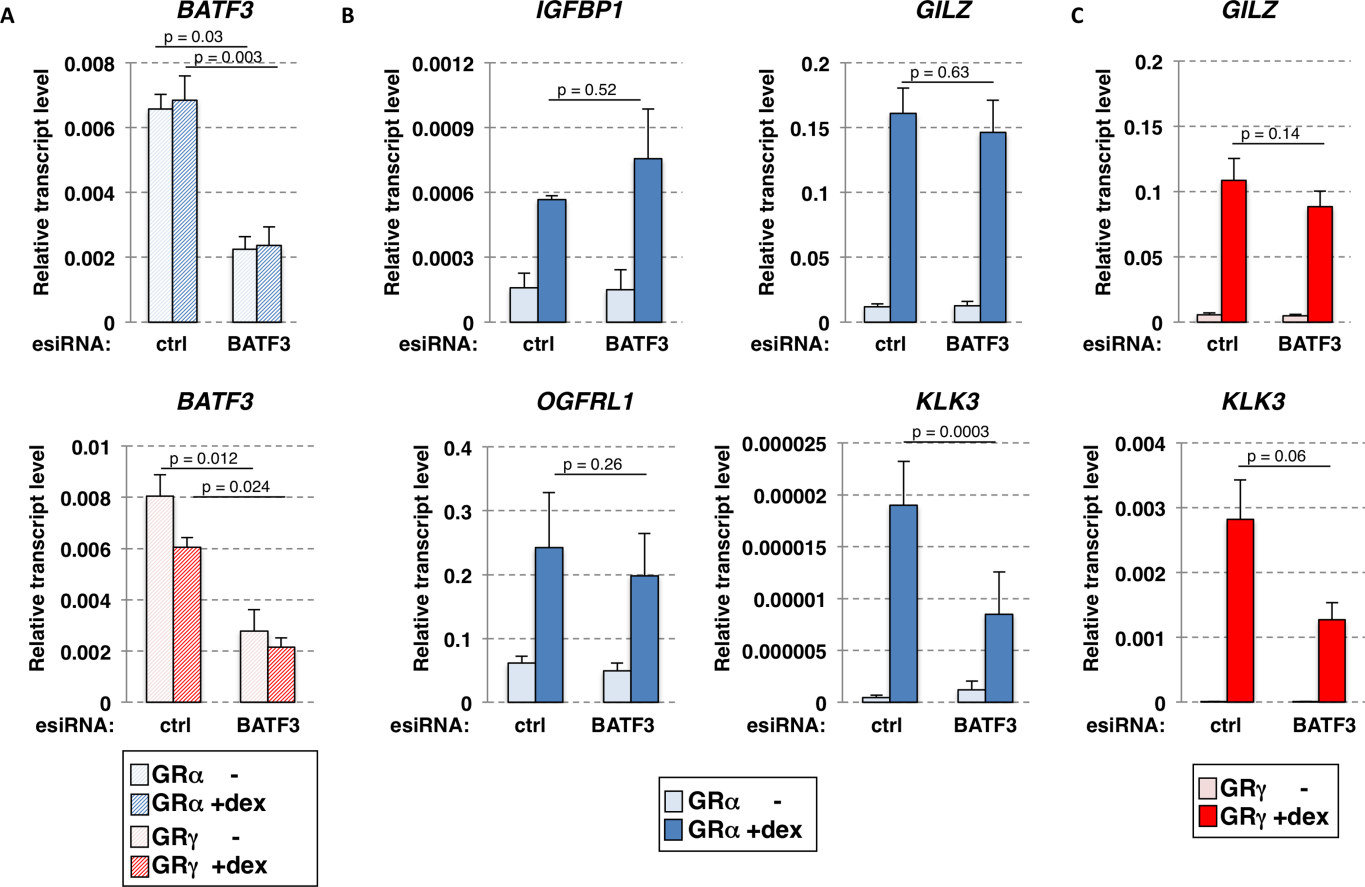
Gene-specific effects of BATF3 knockdown on GRα-dependent transcriptional regulation. (a) Efficacy of esiRNA knockdown of *BATF3*. Relative *BATF3* RNA levels were quantified by qPCR, 52 h after transfection with esiRNAs targeting *BATF3* or using a non-target control for both treated (4h, 1 μM dex) and untreated (ethanol vehicle -) U2OS cells stably expressing either GRα (top) or GRγ (bottom). Averages ± SEM (n = 3) are shown. (b) Same as for (a) except that the relative expression of GR target genes as indicated was quantified by qPCR for U2OS cells stably expressing GRα. (c) Same as for (b) except that U2OS cells stably expressing GRγ were analyzed. (a-c) The p-values were calculated using a paired two-tailed Student’s t-test (n = 3).

To test the role of endogenously expressed BATF3 in GR-dependent gene regulation, we knocked down *BATF3* expression using esiRNAs in U2OS cells stably expressing either GRα or GRγ. qPCR analysis indicated that *BATF3* mRNA levels were reduced by approximately 65% (Fig 4A). Next, we assayed the effect of the knockdown on a panel of GR-regulated genes in cells expressing GRα. These genes included a gene that is regulated similarly by both GRα and GRγ (*GILZ*), two genes that are regulated more robustly by GRα than GRγ (*IGFBP1* and *OGFRL1*) and a gene that is regulated more robustly by GRγ than GRα (*KLK3*) [22]. We found that knockdown of *BATF3* did not have an obvious effect on the GRα-dependent regulation of *GILZ*, *IGFBP1* and *OGFRL1* (Fig 4B). In contrast, GRα-dependent regulation of *KLK3* was reduced by ~ 50% upon reduction of BATF3 levels (Fig 4B). Similarly, knockdown of *BATF3* did not have an obvious effect on the GRγ-dependent regulation of *GILZ* whereas GRγ-dependent regulation of *KLK3* was reduced by ~ 50% upon reduction of *BATF3* levels (Fig 4C). Together, the knockdown experiments corroborate our findings with luciferase reporters that BATF3 can act as a coactivator of both GR isoforms in a context (gene) specific manner. Furthermore, they indicate that the isoform-specific interaction we observe in our co-IP assays does not appear to result in an isoform-specific ability of BATF3 to function as a GR coactivator.

### GBS-specific coactivator activity of BATF3

**Fig 5:**
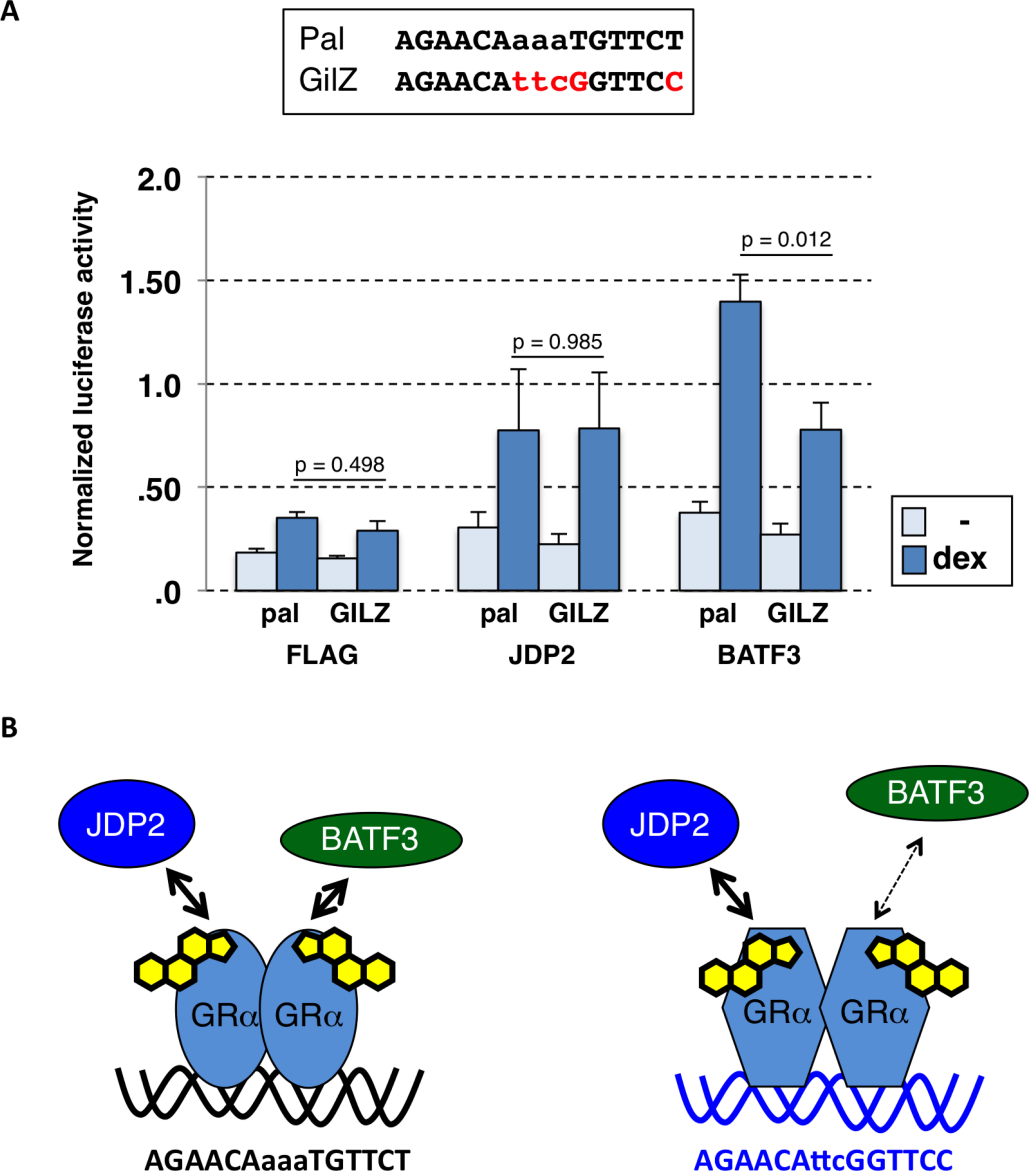
Strength of BATF3-dependent modulation of GRα activity varies between GBSs. (a) U2OS cells were cotransfected with an expression construct for GRα, a luciferase reporter plasmid with either the pal or GILZ GBS and an expression construct for either JDP2, BATF3 or FLAG-tag only as control. Average normalized luciferase ± SEM (n≥3) is shown for both treated (overnight, 1 μM dex) and untreated (ethanol vehicle, -) cells. The p-values were calculated using a two-tailed Student’s t-test (n≥3). (b) Cartoon depicting the co-activator role of JDP2 that is non-differential between two GBS variants, whereas the co-activator role of *BATF3* quantitatively differs between GBS variants.

The context-specific effects of BATF3 could be a consequence of differences in core promoters, chromatin context, distinct transcription factor binding site composition and variation in the DNA sequence bound by GR. To test if the effect of BATF3 is GBS-specific, we compared its effect on a panel of GBS-reporters [16,17] that are identical except for the sequence of the GR binding site. For each of the GBS-variants tested, we found that overexpression of BATF3 resulted in increased GRα-dependent activation of the reporter (data not shown). Interestingly however, the magnitude of the effect varied between GBS variants. Most notably, when we compared two reporters with comparable activities in the absence of overexpressed BATF3 (pal and GILZ, Fig 5A) we found that the hormone-induced levels upon BATF3 overexpression were almost twice as high than those of the GILZ GBS (Fig 5A). Given the sequence similarity between *BATF3* and *JDP2*, we also tested the effect of JDP2 overexpression on these two GBS variants. Consistent with previous studies [28], we found that JDP2 can act as a GR co-activator of GRα. However, in contrast to BATF3 the effect of JDP2 was comparable for the pal and GILZ GBSs (Fig 5A). Together, these results suggest that variation in the GBS can direct context-specific coactivator potency of BATF3 (Fig 5B).

## Discussion

Glucocorticoids are released in response to various kinds of stress. Depending on the type of stress, different physiological responses to glucocorticoids occur to maintain homeostasis. For example, glucocorticoids released upon starvation promote glucose synthesis in the liver to maintain normal blood glucose levels whereas glucocorticoids released in response to sepsis play a role in suppressing immunological responses to prevent them from becoming pathological [32,33]. The distinct physiological responses to glucocorticoids are likely a consequence of GR’s ability to regulate distinct sets of genes in different cell types. One explanation for the limited overlap in the genes regulated by GR is that the genomic loci bound by GR vary between cell types [34,35]. Tissue-specific actions of glucocorticoids may also result from the tissue-specific expression of GR isoforms that regulate distinct sets of genes [20,22]. Our study identifies BATF3 as a coactivator of GR whose coregulatory potential differs between GR isoforms suggesting that the interplay between GR isoform and BATF3 expression could contribute to the context-specific transcriptional consequences of GR signaling. BATF3 is a transcription factor from the AP1 family that plays a critical role in the development of classical dendritic cells [36], which are implicated in the adaptive immune response. However, the expression of BATF3 is not restricted to the immune system. Other tissues expressing BATF3 include the adrenal gland and lung [37] thus arguing that BATF3 may serve as a GR coactivator in several cell types. Our Y2H screen also uncovered isoform-invariant interactions between GR and macroH2A2, a histone variant associated with repressed chromatin (Fig 1D), and interactions between GR and histones H3 and H2A (data not shown). The interaction of GR with core histones has been reported by others [38] and might be relevant for GR’s ability to bind to inaccessible chromatin regions [39]. For example, GR binding to inaccessible chromatin might occur via partial recognition of the GBS displayed on the nucleosome surface and added affinity might come from GR-histone interactions as has been proposed for pioneer transcription factors involved in reprogramming [40].

Several assays we performed indicated that the strength of interaction with BATF3 differs between GRα and GRγ. The Y2H assay suggests a stronger interaction of BATF3 with GRγ than GRα. In contrast, the co-IP assays show that the interaction with BATF3 is stronger for GRα than GRγ. The context-dependent modulation of the interaction by changes in the lever arm may be explained by differences between these assays. Specifically, GR is presumably not DNA-bound in the co-IP assay, which uses cleared protein extracts. Conversely, activation in the Y2H assay depends on the interaction of the LexA DNA binding domain of the fusion protein with LexA DNA binding sites. Other possible explanations for the differences between the Y2H and co-IP assays include that GR and BATF3 are fused to distinct domains for each assay. Further, the AF1 domain is mutated for the Y2H assay and not for the co-IP assays. Finally, the Y2H assays were performed in yeast whereas the co-IP assays were done using mammalian cells, which might account for the observed differences.

Notably, structural studies indicate that the lever arm undergoes a major structural rearrangement in response to DNA binding [41]. Further, NMR data indicates that changes in the lever arm are propagated to other parts of the DBD including helix 3 [22] and that both helix 3 and the lever arm are involved in interactions between JDP2 and PR, close homologs of BATF3 and GR respectively [29]. Thus, a possible explanation for the context-specific effect of changing the lever arm might be that the arginine insertion for GRγ stabilizes the interaction between GR and BATF3 in certain contexts whereas the interaction is weakened by the insertion in other conditions. For example, conformational changes induced in the lever arm by variations in the DNA sequence bound by GR could either strengthen or weaken the interaction between BATF3 and GR and thereby explain the GBS-specific (Fig 5A) and possibly gene-specific (Fig 4) activities of BATF3 as a coactivator. Studies with PR have shown that JDP2 is recruited to the DNA by PR [28] and that it forms a ternary complex with PR on DNA *in vitro* [28]. Thus, one possible explanation for the *KLK3*-specific effect of BATF3 knockdown could be that GR adopts a BATF3 interaction-competent conformation on binding sites responsible for the regulation of *KLK3* whereas this is not the case for GR binding sites associated with other GR-regulated genes we tested. Similarly, *KLK3*-specific direct DNA binding by BATF3 could explain the GR target gene-specific effect of BATF3 knockdown. To test if the gene-specific effects of BATF3 knockdown might be explained by target-gene specific recruitment to the *KLK3* gene, we performed ChIP experiments targeting BATF3. However, we failed to observe convincing GR-dependent BATF3 recruitment to GR binding sites near the *KLK3* gene or to any of the other GR-bound loci we examined. Interpretation of these results however is problematic in the absence of a positive control. Thus, the absence of BATF3 recruitment could either reflect technical difficulties or a lack of recruitment which could indicate that BATF3’s function as GR-coactivator might differ mechanistically from JDP2’s coactivator role for PR. Arguing for the later possibility, previous studies have shown that JDP2 induces structural changes in the N-terminal transcription activation function 1 (AF1) domain of both GR and PR [27,42] a domain which interacts with a variety of coregulators. In contrast, the BATF3-induced changes in the N-terminal AF1 domain of PR are only minimal [42]. Further, we observed that JDP2, in contrast to BATF3, interacts equally strong with both GRα and GRγ (Fig 2) and that the coactivator potency for JDP2 is comparable for GBS variants (Fig 5).

In summary, we identify BATF3 as a context-specific coactivator of GR whose interaction with GR differs between GR isoforms GRα and GRγ. However, despite the isoform-specific physical interaction, BATF3 appears to act as coactivator for both isoforms. In addition, we find that sequence variation in the DNA binding site of GR influences the coactivator potential of BATF3 (Fig 5B). Interestingly, the conformational changes induced by changes in DNA sequence and by the arginine insertion in the lever arm show extensive overlap and might thus modulate the interaction via similar mechanism [22]. However, a detailed understanding of the mechanisms that modulate the interaction between BATF3 and GR and of how BATF3 potentiates GR-dependent regulation in a context-specific manner will require additional studies including structural analysis of full length GR in complex with BATF3. We speculate that this involves changes in the interface of GR interacting with BATF3 and that this might contribute the gene-specific and tissue-specific consequences of GR signaling.

## Materials and methods

### Yeast two-hybrid analysis

Yeast two-hybrid screening was performed essentially as described [43] with minor modifications. Briefly, full length rat GRα and GRγ, with mutations in the AF1 domain (E219K/F220L/W234R) to reduce auto-activation by the bait, were cloned into the pBTM116-D9 vector using primers as listed in Table 1. Screens were done in the absence of hormone, and in the presence of either 0.5 μM or 1 μM desoxycorticosterone (Fischer Scientific, CA164435000) for GRα or 0.25 μM or 0.5 μM desoxycorticosterone for GRγ. Prey strains interacting with both GR isoforms and preys with isoform-specific interactions were retested using fresh yeast cells and the identity of interaction partners was verified by sequencing.

**Table 1:** Primers used for cloning.

Rat GR-fw:	AAAAAGCAGGCTTAATGGACTCCAAAGAATCCTTAGC
Rat-GR-rev:	AGAAAGCTGGGTCTCATTTTTGATGAAACAGAAGC
hJDP2-fw-attB	AAAAAGCAGGCTTAATGGTAGCAGGCTGGCCTGCC
hJDP2 rev-attB	AGAAAGCTGGGTCTCACTTCTTCTCGAGCTGCTCGAG

Underlined part targets rGR/JDP2; 5’ end required for gateway cloning.

### Co-immunoprecipitation (Co-IP) assays in mammalian cells

Co-IP assays were essentially done as described [30]. In short, ORFs were transferred to firefly‐V5 fusion vectors (pcDNA3.1V5Fire‐DM; “firefly‐tag”) and to protein A fusion vectors (pcDNA3.1PA‐D57; “PA‐tag”) using standard Gateway cloning procedures. Primers for cloning JDP2 are listed in Table 1. For luciferase‐based co‐IP assays, 30.000 T-Rex 293 cells were seeded in a well of a 96-well plate and transfected the following day with 50ng each of PA and firefly-tag expression constructs using Lipofectamine 2000 (Invitrogen). The next day, cells were either treated for 1 h with vehicle (ethanol), or 1 μM dexamethasone before cells were lyzed for 30 min at 4°C in 100 μl lysis buffer (50 mM Hepes (pH 7.4); 150 mM NaCl; 1 mM EDTA; 10% glycerol; 1% Triton X‐100; 1% phosphatase inhibitor cocktail 2 (Sigma‐Aldrich, P5726), protease inhibitor (Roche, 11836170001) with either vehicle or 1 μM dexamethasone. Protein complexes were precipitated from 70 μl cleared cell extract in IgG‐coated microtiter plates for 1 h at 4°C and washed three times with 100 μl ice‐cold PBS. The binding of the firefly‐V5‐tagged fusion protein (co‐IP) to the PA‐tagged fusion protein (IP) was assessed by measuring the firefly luciferase activity in a luminescence plate reader (LUMIstar, BMG Labtech using the Bright‐Glo Luciferase Assay (Promega)). Luciferase activity was normalized to input and the fold change over the activity observed for protein A was calculated and averaged from transfections performed at least in triplicate.

### Immunoblotting

Cleared lysates from cells transfected for co-IP experiments or whole-cell lysates from cells transfected for luciferase assays were separated with SDS/PAGE gels, transferred to nitrocellulose membranes, and incubated with either anti-GR (N499, 1:3000), anti-FLAG (M2, F1804; Sigma-Aldrich, 1:500) or anti-actin (Sc-1616R; Santa Cruz Biotechnology, 1:1000) antibodies followed by incubation with secondary antibodies conjugated with horseradish peroxidase. Proteins were visualized using the SuperSignal West Dura substrate (ThermoFisher).

### Luciferase assays

Transient transfections were done essentially as described [17]. Luciferase reporter constructs pGILZ [31], CGT-luc, pal-luc and GILZ-luc [17] have been described previously. Expression constructs for JDP2 and BATF3 were generated by shuttling ORFs for these genes to Gateway destination vector pFLAG-CMV-D11. Briefly, U2OS cells, U2OS cells stably expressing GRα or U2OS cells stably expressing GRγ were seeded into 48-well plates in DMEM-5% FBS at approximately 30,000 cells per well and transfected the following day in FBS-free DMEM using 0.4 μl of Lipofectamine and 0.8 μl of PLUS reagent (Invitrogen) per well according to manufacturer's instructions. Per well, a total of 85 ng of DNA was transfected consisting of 5 ng each of GR expression construct (left out for U2OS cells stably expressing GRα or GRγ) and luciferase reporter, 0.05 ng of pRL renilla, the indicated amount of expression construct for BATF3 or JDP2 plus empty expression plasmid to a total of 30ng and 45 ng of the plasmid p6R. Cells were treated overnight with 1 μM dexamethasone, harvested and luciferase activity was measured using the dual luciferase assay kit (Promega).

### EsiRNA knockdown

For esiRNA knockdown experiments of BATF3, 10.000 U2OS cells stably expressing GRα [44] or GRγ [22] were seeded per well of a 48-well plate. The next day, cells were transfected with 50nM esiRNAs (Sigma-Aldrich) against BATF3 (EHU153831) or a non-target esiRNA control (RL, EHURLUC) using lipofectamine 2000 (Invitrogen). 6 h after transfection, cells were washed once and re-fed with DMEM/5% FBS. 48 h past transfection, cells were treated for 4 h with 1 μM dexamethasone or ethanol as vehicle control to measure the effect of BATF3 knockdown on GR-dependent regulation of endogenous target genes. RNA was isolated using an RNeasy kit (Qiagen) with on-column DNAse digestion prior to reverse transcription and analysis by Quantitative Real Time PCR using the primers listed in Table 2.

**Table 2:** Primers used for qPCR analysis.

Gene/Locus:	Fw primer:	Rev. Primer:
*RPL19*	ATGTATCACAGCCTGTACCTG	TTCTTGGTCTCTTCCTCCTTG
*BATF3*	GGAGCAAGAAAACACCATGC	CATCTTCTCGTGCTCCTTCAG
*IGFBP1*	TCACAGCAGACAGTGTGAGAC	AGACCCAGGGATCCTCTTC
*GILZ*	AGATCGAACAGGCCATGGAT	TTACACCGCAGAACCACCAG
*OGFRL1*	CCACTGAAGCAACTGCCAAA	CACCATCTGGCTTGAATGGA
*KLK3*	AGCATTGAACCAGAGGAGTTCT	CCCGAGCAGGTGCTTTTG
